# Supplementary material for: A G358S mutation in the Plasmodium falciparum Na+ pump PfATP4 confers clinically-relevant resistance to cipargamin
Source: Nat Commun. 2022 Sep 30;13:5746. doi: 10.1038/s41467-022-33403-9 (PMC9525273; doi:10.1038/s41467-022-33403-9)
Supplement: Supplementary file 1 — Supplementary Information PDF file [file 41467_2022_33403_MOESM1_ESM.pdf]

A G358S mutation in the *Plasmodium falciparum* Na^+ pump PfATP4 confers clinically-relevant resistance to cipargamin

Supplementary Information

Contains:

Supplementary Tables 1-7

Supplementary Note

Supplementary Figures 1-10

Supplementary References

Supplementary Table 1. Susceptibility of the *P. falciparum* lines generated through *in vitro* evolution in this study and their parents to antiplasmodial compounds. The IC_{50} values (mean ± SEM) for growth inhibition for the parasite lines and compounds indicated are shown, with the number of independent experiments (performed on different days) shown in brackets. Within each parasite set, all lines were tested in parallel in each experiment. For each compound, the IC_{50} values for each cipargamin-resistant line were compared with those of its direct parent (Dd2 for Dd2-PfATP4^{T418N,P990R}; Dd2-PfATP4^{T418N,P990R} for HCR1 and HCR2; and Dd2-Polδ for Dd2-Polδ-PfATP4^{G358S}) using two-tailed paired t-tests. *P* values ≤ 0.05 indicate statistical significance and are shown in bold. Source data are provided as a Source Data file.

	Mean IC_{50} value ± SEM in nM					
	Set 1				Set 2	
	Dd2	Dd2-PfATP4^{T418N,P990R}	HCR1	HCR2	Dd2-Polδ	Dd2-Polδ-PfATP4^{G358S}
Cipargamin	0.68 ± 0.03 (16)	9.17 ± 0.46 (16) **P = 5×10^{-12}**	2840 ± 190 (16) **P = 2×10^{-10}**	2920 ± 200 (16) **P = 3×10^{-10}**	1.43 ± 0.20 (7)	1420 ± 100 (7) **P = 7×10^{-6}**
(+)-SJ733	60 ± 2 (5)	331 ± 11 (5) **P = 9×10^{-6}**	21500 ± 2400 (5) **P = 0.0009**	17600 ± 2000 (5) **P = 0.001**	40.7 ± 4.4 (4)	9070 ± 1010 (4) **P = 0.003**
PA21A050	1.20 ± 0.04 (3)	2.70 ± 0.16 (3) **P = 0.01**	61 ± 10 (3) **P = 0.03**	60 ± 4 (3) **P = 0.005**	2.42 ± 0.24 (4)	12.9 ± 1.2 (4) **P = 0.002**
Chloroquine	127 ± 21 (4)	131 ± 11 (4) P = 0.7	115 ± 16 (4) P = 0.06	167 ± 21 (4) **P = 0.03**	185 ± 35 (5)	182 ± 27 (5) P = 0.8
Dihydro-artemisinin	2.17 ± 0.28 (4)	2.44 ± 0.23 (4) P = 0.1	1.55 ± 0.21 (4) **P = 0.005**	1.51 ± 0.12 (4) **P = 0.05**	2.15 ± 0.21 (4)	2.58 ± 0.27 (4) P = 0.4
MMV006656	Not tested				344 ± 43 (4)	1800 ± 230 (4) **P = 0.01**
MMV665949	Not tested				4270 ± 620 (4)	4870 ± 880 (4) P = 0.4

Supplementary Table 2. Whole-genome sequence metrics of the NF54^{WT} parent strain, the *pfatp4* gene-edited control (NF54^{CTL}) and two independently generated G358S mutant parasite lines, NF54$^{G358S-1}$ and NF54$^{G358S-2}$. The genome-wide mean fold coverage (in bold) was 24 to 39 across all samples.

		Gene-edited NF54 lines			Parent
**Sample names**		**NF54^{CTL}**	**NF54$^{G358S-1}$**	**NF54$^{G358S-2}$**	**NF54^{WT}**
**Total reads**		4,006,427	4,057,217	3,100,427	4,358,465
**# Mapped reads**		3,803,391	3,826,201	2,470,553	4,113,893
**Duplication rate**		26.7%	28.5%	32.1%	38.9%
**General error rate**		0.6%	0.6%	0.5%	0.6%
**Mean mapping quality (Phred)**		59.2	59.3	59.0	59.3
**Depth of coverage**	**mean**	**39.3**	**39.0**	**24.2**	**39.0**
	**SD**	27.5	26.6	135.3	26.0
**% of Pf genome with > x no. reads**	**1X**	99.1%	99.1%	98.0%	99.0%
	**5X**	98.5%	98.2%	82.2%	98.2%
	**10X**	96.5%	95.8%	53.3%	96.1%
	**30X**	71.3%	70.1%	12.2%	72.0%

Supplementary Table 3. High confidence, non-synonymous and synonymous SNPs in coding regions called from whole-genome sequencing analyses of the *pfatp4* gene-edited control and G358S mutant parasite lines compared to the NF54^{WT} parent strain. These include the 17 silent binding-site mutations introduced into the *pfatp4* locus.

CHROM	POS	REF	ALT	AMINO ACID CHANGE	CODON CHANGE	GENE NAME	EFFECT / IMPACT
Pf3D7_03_v3	201218	A	G	V78A	gTt/gCt	PF3D7_0304000 (inner membrane complex protein 1a, putative)	NON SYN CODING / MODERATE
Pf3D7_12_v3	2101412	A	T	N521Y	Aat/Tat	PF3D7_1251500 (ATP-dependent RNA helicase DRS1, putative)	NON SYN CODING / MODERATE
Pf3D7_12_v3	531728	C	T	G358S	Ggt/Agt	PF3D7_1211900 (non-SERCA-type Ca2 - transporting P-ATPase)	NON SYN CODING / MODERATE
Pf3D7_12_v3	531729	T	C	L357	ttA/ttG	PF3D7_1211900 (non-SERCA-type Ca2 - transporting P-ATPase)	SYN CODING / LOW
Pf3D7_12_v3	531738	T	C	L354	ttA/ttG	PF3D7_1211900 (non-SERCA-type Ca2 - transporting P-ATPase)	SYN CODING / LOW
Pf3D7_12_v3	531744	T	A	V352	gtA/gtT	PF3D7_1211900 (non-SERCA-type Ca2 - transporting P-ATPase)	SYN CODING / LOW
Pf3D7_12_v3	531756	T	A	T348	acA/acT	PF3D7_1211900 (non-SERCA-type Ca2 - transporting P-ATPase)	SYN CODING / LOW
Pf3D7_12_v3	531780	T	C	K340	aaA/aaG	PF3D7_1211900 (non-SERCA-type Ca2 - transporting P-ATPase)	SYN CODING / LOW
Pf3D7_12_v3	531792	G	T	S336	tcC/tcA	PF3D7_1211900 (non-SERCA-type Ca2 - transporting P-ATPase)	SYN CODING / LOW
Pf3D7_12_v3	531801	T	C	K333	aaA/aaG	PF3D7_1211900 (non-SERCA-type Ca2 - transporting P-ATPase)	SYN CODING / LOW
Pf3D7_12_v3	531807	T	G	V331	gtA/gtC	PF3D7_1211900 (non-SERCA-type Ca2 - transporting P-ATPase)	SYN CODING / LOW
Pf3D7_12_v3	531810	T	C	Q330	caA/caG	PF3D7_1211900 (non-SERCA-type Ca2 - transporting P-ATPase)	SYN CODING / LOW
Pf3D7_12_v3	531813	T	C	T329	acA/acG	PF3D7_1211900 (non-SERCA-type Ca2 - transporting P-ATPase)	SYN CODING / LOW
Pf3D7_12_v3	531828	G	T	S324	tcC/tcA	PF3D7_1211900 (non-SERCA-type Ca2 - transporting P-ATPase)	SYN CODING / LOW
Pf3D7_12_v3	531834	A	G	V322	gtT/gtC	PF3D7_1211900 (non-SERCA-type Ca2 - transporting P-ATPase)	SYN CODING / LOW
Pf3D7_12_v3	531837	A	G	I321	atT/atC	PF3D7_1211900 (non-SERCA-type Ca2 - transporting P-ATPase)	SYN CODING / LOW
Pf3D7_12_v3	531843	T	C	K319	aaA/aaG	PF3D7_1211900 (non-SERCA-type Ca2 - transporting P-ATPase)	SYN CODING / LOW
Pf3D7_12_v3	531846	A	G	G318	ggT/ggC	PF3D7_1211900 (non-SERCA-type Ca2 - transporting P-ATPase)	SYN CODING / LOW
Pf3D7_12_v3	531849	A	C	S317	tcT/tcG	PF3D7_1211900 (non-SERCA-type Ca2 - transporting P-ATPase)	SYN CODING / LOW
Pf3D7_12_v3	531861	T	G	V313	gtA/gtC	PF3D7_1211900 (non-SERCA-type Ca2 - transporting P-ATPase)	SYN CODING / LOW

Supplementary Table 4. High confidence, non-synonymous and synonymous SNPs in coding regions and their allele frequencies called from whole-genome sequencing analyses for each of the *pfatp4* gene-edited control and G358S mutant parasite lines compared to the NF54^{WT} parent strain. The G358S mutation in PfATP4 associated with resistance to cipargamin and (+)-SJ733 was found exclusively in the NF54$^{G358S-1}$ and NF54$^{G358S-2}$ mutant lines, in addition to the 17 silent binding-site mutations at the guide RNA cleavage site within the *pfatp4* locus that were also introduced in the *pfatp4* gene-edited control line (NF54^{CTL}).

GENE NAME	AMINO ACID CHANGE	CODON CHANGE	Gene-edited NF54 parasite lines		
			NF54^{CTL}	NF54$^{G358S-1}$	NF54$^{G358S-2}$
PF3D7_0304000	V78A	gTt/gCt	✓ [90%]		✓ [100%]
PF3D7_1251500	N521Y	Aat/Tat		✓ [100%]	
PF3D7_1211900	G358S	Ggt/Agt		✓ [100%]	✓ [100%]
PF3D7_1211900	L357	ttA/ttG	✓ [100%]	✓ [100%]	✓ [100%]
PF3D7_1211900	L354	ttA/ttG	✓ [100%]	✓ [100%]	✓ [100%]
PF3D7_1211900	V352	gtA/gtT	✓ [100%]	✓ [100%]	✓ [100%]
PF3D7_1211900	T348	acA/acT	✓ [100%]	✓ [100%]	✓ [100%]
PF3D7_1211900	K340	aaA/aaG	✓ [100%]	✓ [100%]	✓ [100%]
PF3D7_1211900	S336	tcC/tcA	✓ [100%]	✓ [100%]	✓ [100%]
PF3D7_1211900	K333	aaA/aaG	✓ [100%]	✓ [100%]	✓ [100%]
PF3D7_1211900	V331	gtA/gtC	✓ [100%]	✓ [100%]	✓ [100%]
PF3D7_1211900	Q330	caA/caG	✓ [100%]	✓ [100%]	✓ [100%]
PF3D7_1211900	T329	acA/acG	✓ [96%]	✓ [100%]	✓ [100%]
PF3D7_1211900	S324	tcC/tcA	✓ [100%]	✓ [100%]	✓ [100%]
PF3D7_1211900	V322	gtT/gtC	✓ [100%]	✓ [100%]	✓ [100%]
PF3D7_1211900	I321	atT/atC	✓ [100%]	✓ [100%]	✓ [100%]
PF3D7_1211900	K319	aaA/aaG	✓ [100%]	✓ [100%]	✓ [100%]
PF3D7_1211900	G318	ggT/ggC	✓ [100%]	✓ [100%]	✓ [100%]
PF3D7_1211900	S317	tcT/tcG	✓ [100%]	✓ [100%]	✓ [100%]
PF3D7_1211900	V313	gtA/gtC	✓ [100%]	✓ [100%]	✓ [100%]

✓ denotes SNPs found in the edited NF54 parasite lines; mutant allele frequencies are indicated within square brackets.

Supplementary Table 5. Susceptibility of the NF54-based lines to antiplasmodial compounds. IC_{50} and IC_{90} values (mean ± SEM; nM) for two independently-generated PfATP4 G358S mutant NF54 parasite lines and their controls are shown. The *pfatp4* genotype, mean IC_{90}/IC_{50} ratios ± SEM, and fold-changes (FC) in the IC_{50} and IC_{90} values, and the IC_{90}/IC_{50} ratios for each edited line compared to the NF54 parental strain are also shown. Source data are provided as a Source Data file.

Parasite line	*pfatp4*	N[c]	IC_{50} ± SEM (nM)	IC_{90} ± SEM (nM)	Mean IC_{90}/IC_{50} ± SEM	FC[d] (IC_{50})	FC[d] (IC_{90})	FC[d] (Mean IC_{90}/IC_{50})
				Cipargamin				
$NF54^{WT}$	WT[a]	4	2.6 ± 0.1	3.8 ± 0.3	1.4 ± 0.1	1.0	1.0	1.0
$NF54^{CTL}$	WT+bsm[b]	4	2.6 ± 0.1	3.7 ± 0.2	1.4 ± 0.03	1.0 (0.3)	1.0 (0.7)	1.0 (1.0)
$NF54^{G358S-1}$	G358S+bsm	4	2029 ± 93.9	3461 ± 30.7	1.7 ± 0.1	768.6 (**0.0002**)	910.8 (**2×10^{-6}**)	1.2 (0.09)
$NF54^{G358S-2}$	G358S+bsm	4	1985 ± 161.2	3433 ± 36.7	1.8 ± 0.1	751.9 (**0.001**)	903.4 (**3×10^{-6}**)	1.2 (**0.05**)
				(+)-SJ733				
$NF54^{WT}$	WT[a]	4	93.8 ± 4.4	163.5 ± 7.8	1.7 ± 0.1	1.0	1.0	1.0
$NF54^{CTL}$	WT+bsm[b]	4	84.8 ± 1.8	150.5 ± 6.9	1.8 ± 0.1	0.9 (0.2)	0.9 (0.4)	1.0 (0.8)
$NF54^{G358S-1}$	G358S+bsm	4	13959 ± 682.8	25415 ± 726.5	1.8 ± 0.1	148.8 (**0.0003**)	155.4 (**5×10^{-5}**)	1.1 (0.7)
$NF54^{G358S-2}$	G358S+bsm	4	12955 ± 479.7	24998 ± 892.1	1.9 ± 0.1	138.1 (**0.0001**)	152.9 (**0.0001**)	1.1 (0.3)
				Dihydroartemisinin (DHA)				
$NF54^{WT}$	WT[a]	4	0.8 ± 0.1	2.3 ± 0.3	2.8 ± 0.2	1.0	1.0	1.0
$NF54^{CTL}$	WT+bsm[b]	4	0.9 ± 0.1	2.7 ± 0.1	3.1 ± 0.2	1.1 (0.6)	1.2 (0.3)	1.1 (0.1)
$NF54^{G358S-1}$	G358S+bsm	4	0.9 ± 0.1	2.6 ± 0.1	2.9 ± 0.2	1.1 (0.4)	1.1 (0.4)	1.1 (0.3)
$NF54^{G358S-2}$	G358S+bsm	4	0.9 ± 0.1	2.8 ± 0.1	3.2 ± 0.4	1.1 (0.5)	1.2 (0.1)	1.2 (0.2)
				INE963				
$NF54^{WT}$	WT[a]	4	0.8 ± 0.1	1.9 ± 0.4	2.4 ± 0.1	1.0	1.0	1.0
$NF54^{CTL}$	WT+bsm[b]	4	1.2 ± 0.1	2.5 ± 0.3	2.1 ± 0.1	1.5 (**0.008**)	1.3 (0.06)	0.9 (0.1)
$NF54^{G358S-1}$	G358S+bsm	4	1.1 ± 0.1	2.7 ± 0.3	2.4 ± 0.1	1.4 (**0.01**)	1.4 (0.08)	1.0 (0.8)
$NF54^{G358S-2}$	G358S+bsm	4	1.1 ± 0.1	2.7 ± 0.3	2.4 ± 0.2	1.4 (**0.01**)	1.4 (**0.02**)	1.0 (1.0)
				KAF156				
$NF54^{WT}$	WT[a]	7	6.7 ± 0.2	13.0 ± 1.0	1.9 ± 0.1	1.0	1.0	1.0
$NF54^{CTL}$	WT+bsm[b]	7	4.9 ± 0.2	10.4 ± 0.8	2.1 ± 0.1	0.7 (**0.0008**)	0.8 (**0.01**)	1.1 (0.2)
$NF54^{G358S-1}$	G358S+bsm	7	5.9 ± 0.4	12.8 ± 1.3	2.1 ± 0.1	0.9 (0.1)	1.0 (0.8)	1.1 (0.09)
$NF54^{G358S-2}$	G358S+bsm	7	5.6 ± 0.4	12.8 ± 1.5	2.2 ± 0.1	0.8 (**0.05**)	1.0 (0.9)	1.2 (**0.02**)
				Pyronaridine				
$NF54^{WT}$	WT[a]	4	1.4 ± 0.3	2.6 ± 0.6	1.9 ± 0.1	1.0	1.0	1.0
$NF54^{CTL}$	WT+bsm[b]	4	1.6 ± 0.1	2.9 ± 0.02	1.8 ± 0.1	1.2 (0.3)	1.1 (0.7)	1.0 (0.7)
$NF54^{G358S-1}$	G358S+bsm	4	1.8 ± 0.2	2.9 ± 0.1	1.7 ± 0.2	1.3 (0.07)	1.1 (0.5)	0.9 (0.5)
$NF54^{G358S-2}$	G358S+bsm	4	1.7 ± 0.2	2.8 ± 0.1	1.7 ± 0.1	1.2 (0.2)	1.1 (0.7)	0.9 (0.5)
				Piperaquine				
$NF54^{WT}$	WT[a]	4	19.7 ± 1.8	29.0 ± 0.6	1.5 ± 0.1	1.0	1.0	1.0
$NF54^{CTL}$	WT+bsm[b]	4	17.3 ± 2.4	34.4 ± 4.5	2.0 ± 0.1	0.9 (0.2)	1.2 (0.3)	1.3 (**0.05**)
$NF54^{G358S-1}$	G358S+bsm	4	17.7 ± 2.3	33.0 ± 3.9	1.9 ± 0.1	0.9 (0.3)	1.1 (0.3)	1.3 (**0.07**)
$NF54^{G358S-2}$	G358S+bsm	4	18.0 ± 2.7	33.3 ± 5.1	1.9 ± 0.1	0.9 (0.4)	1.1 (0.4)	1.2 (**0.07**)
				Monodesethyl-amodiaquine				
$NF54^{WT}$	WT[a]	4	6.9 ± 1.2	9.2 ± 1.8	1.3 ± 0.03	1.0	1.0	1.0
$NF54^{CTL}$	WT+bsm[b]	4	7.0 ± 0.2	13.6 ± 0.1	2.0 ± 0.04	1.0 (1.0)	1.5 (0.09)	1.5 (**0.001**)
$NF54^{G358S-1}$	G358S+bsm	4	7.6 ± 0.8	12.4 ± 1.7	1.6 ± 0.1	1.1 (0.5)	1.4 (0.2)	1.2 (0.09)
$NF54^{G358S-2}$	G358S+bsm	4	6.8 ± 0.3	12.2 ± 1.4	1.8 ± 0.1	1.0 (1.0)	1.3 (0.2)	1.3 (**0.04**)

Lumefantrine								
NF54[WT]	WT[a]	4	57.3 ± 11.9	123.3 ± 10.3	2.3 ± 0.3	1.0	1.0	1.0
NF54[CTL]	WT+bsm[b]	4	45.9 ± 9.0	123.4 ± 9.4	2.9 ± 0.4	0.8 (0.08)	1.0 (1.0)	1.2 (**0.05**)
NF54[G358S-1]	G358S+bsm	4	42.5 ± 4.4	136.8 ± 15.3	3.3 ± 0.3	0.7 (0.2)	1.1 (0.3)	1.4 (**0.02**)
NF54[G358S-2]	G358S+bsm	4	41.1 ± 5.8	121.6 ± 14.4	3.0 ± 0.3	0.7 (0.2)	1.0 (0.9)	1.3 (0.2)

[a] NF54[WT] is the mosquito-transmissible parental line expressing a wild-type (WT) *pfatp4* gene that was used for the CRISPR-Cas9 gene editing experiments to generate the PfATP4 G358S mutant and binding-site mutant (bsm) control parasite lines.

[b] NF54[CTL] is the gene-edited control line that harbors silent binding-site mutations at the guide RNA cleavage site within the *pfatp4* locus.

[c] N, number of independent experiments, each with technical duplicates.

[d] Statistical significance was determined against the NF54[WT] control line using two-tailed paired t-tests (GraphPad Prism version 9). The *P* values are shown in brackets. *P* values ≤ 0.05 indicate statistical significance and are shown in bold.

Supplementary Table 6. Predicted free energy of binding (kcal mol^{-1}) of the highest ranked pose when the search space is constrained to the cavity surrounding residue 358. Results are shown for both cipargamin and (+)-SJ733 with the different PfATP4 models and isoforms.

Structure	Cipargamin		(+)-SJ733	
	WT	G358S	WT	G358S
Open Source	-8.9	-8.5	-8.3	-7.5
Custom	-8.7	-8.3	-7.4	-7.5

Supplementary Table 7. Primers used for *pfatp4* amplification and sequencing in this study, and for the genetic modification of *T. gondii* and *P. falciparum* NF54 parasites.

Primer	Sequence (5' – 3')	Use	Reference
P. falciparum			
1	ATGAGTTCTCAAAATAATAATAAACAGG	Amplification and sequencing	(1)
2	TTAATTCTTAATAGTCATATATTTTCTTCTATATATAACC	Amplification and sequencing	(1)
3	TCACCACAATGTACTGTGTTAAGAAA	Sequencing	(1)
4	ATCCAACAAAAGGTTTTGAACCATGT	Sequencing	(1)
5	TACAAACATGTAGAGAAGCACAAGTT	Sequencing	(1)
6	TATCTCCGTCTTCTACATTATTG	Sequencing	(2)
7	ATGACAGCAATTAATGCAGTTAC	Sequencing	(2)
8	CATGTAGTATATCTGCAACTTTAAC	Sequencing	(2)
9	ATATGAGTGAAGGACCAAT	Sequencing	
10	CTTTAGCTATAGAATGTTCA	Sequencing	
T. gondii			
11	CTGTAGTAGTTATGAATATA	Sequencing	
12	CAACGTGGCCTGAATCGACTGTTTTAGAGCTAGAAATAGCAAG	Q5 mutagenesis	
13	AACTTGACATCCCCATTTAC	Q5 mutagenesis	
14	AGGCTCCAGGCTCACTCCCCTTCAACGTGGCCTGAATCGACTG TCTGGCATGATTGGACTGATCGCCATCTGCGTCCTCATCATCGT CGT	Annealing	
15	ACGACGATGATGAGGACGCAGATGGCGATCAGTCCAATCAT GCCAGACAGTCGATTCAGGCCACGTTGAAGGGGAGTGAGCC TGGAGCCT	Annealing	
16	GGTCGAACTGAAGACGAACG	Sequencing	
17	GTTTGAGCGTACAGTGAAGACG	Sequencing	
***P. falciparum* gene editing**			
18	AAGTATATAATATTACAGGTTTAGATACACAAGT	gRNA 1 In-Fusion forward	
19	GCATAGCTCTTAAACACTTGTGTATCTAAACCTGT	gRNA 1 In-Fusion reverse	
20	AAGTATATAATATTGTAACTAGCGGTTCTGGTAA	gRNA 2 In-Fusion forward	
21	GCATAGCTCTTAAACTTACCAGAACCGCTAGTTAC	gRNA 2 In-Fusion reverse	
22	AGAGGTACCGAGCTCGAATTCCTTGAATGCTTGCTTAGCAAC	*pfatp4* donor In-Fusion fwd	
23	CGAAAAGTGCCACCTGACGTCTCGCTAGTTAATTCATTAGAATC	*pfatp4* donor In-Fusion reverse	
24	ACCTTGAATGCTTGCTTAGCAACC	*pfatp4* blood PCR forward; sequencing	
25	GGGTACTTCTATCAAGTAATCTATCAGGTGC	*pfatp4* blood PCR reverse; sequencing	
26	GCATCCTAAAGTTTCAACAGCTGGTAG	*pfatp4* sequencing	

Supplementary Note. Whole-genome sequencing results for HCR1 and HCR2 parasites, their Dd2-PfATP4^{T418N,P990R} parent, and its Dd2 parent.

Four clonal samples sequenced for this study:

Dd2 (parent of Dd2-PfATP4^{T418N,P990R})

Dd2-PfATP4^{T418N,P990R} (parent of HCR1 and HCR2)

HCR1 (Highly Cipargamin Resistant clone 1)

HCR2 (Highly Cipargamin Resistant clone 2)

Sample	coverage when aligned to Dd2 reference	coverage when aligned with 3D7 reference
Dd2	212.8	229.5
Dd2-PfATP4^{T418N,P990R}	192.9	208.2
HCR1	118.6	129.7
HCR2	228.9	249.0

Dd2 alignment rates: 86% - 87%; 3D7 alignment rates: 98%

All subsequent bioinformatics analysis uses reads aligned to PlasmoDB-29_Pfalciparum3D7 reference genome (https://plasmodb.org/plasmo/app/downloads/release-29/Pfalciparum3D7/).

Copy Number Analysis was performed using QDNAseq with 5 and 1 kbp bins using the recommended workflow. Binned counts were filtered by min-mappability=50%, and a blacklist of centromere and telomere regions, then a loess fit used to correct for GC and mappability, and counts converted to copy numbers. They were inspected as copy numbers and as scaled relative to the parent strain.

Dd2-PfATP4^{T418N,P990R} and Dd2 both had the same amplification on chromosome 5. Scaled, relative copy numbers showed no features of interest. HCR1 and HCR2 copy numbers scaled relative to Dd2-PfATP4^{T418N,P990R} both showed a duplication on chromosome 12, and HCR2 had a relative deletion on chromosome 5, showing a reduction in the observed amplification (Supplementary Fig. 1).

Structural variation analysis used GRIDSS, with candidate SVs inspected in Integrated Genome Viewer (IGV), found no new structural variations in Dd2-PfATP4^{T418N,P990R} compared to Dd2. There was a clear event in chromosome 12 in

both HCR1 and HCR2 (Supplementary Fig. 1). This is a duplication from 520kb – 556kb, covering nine genes including PF3D7_1211900, which is *pfatp4*. The breakends are in homopolymer runs of A, at Pf3D7_12_v3:520014..520039 and Pf3D7_12_v3:556464..556489.

Single-nucleotide changes and small insertions and deletions were called with SNVer. VarScan was also run but did not give usable results. All calls which also appeared in the parent strain were discarded, and remaining calls filtered by depth at least 10, alternate-frequency > 0.4, discard calls in the first and last 10% of each chromosome. When calls are filtered for 'in a coding sequence', ignore genes described as PfEMP1, rifin, stevor or pseudogene. This left 100s of SNPs and 10s of indels. On inspection most of these events were clustered in areas of low coverage, or highly repetitive regions, and some of the remainder were synonymous changes.

Small variants of possible interest

Sample	Position	AltCount/ Depth	Change	Gene	Comment or description
Dd2-PfATP4$^{T418N, P990R}$	chr12: 529831	108/108	G->C; P->R	PF3D7_1211900	Known P990R in PfATP4
Dd2-PfATP4$^{T418N, P990R}$	chr12: 531547	102/102	G->T; T->N	PF3D7_1211900	Known T418N in PfATP4
HCR1, HCR2	chr12: 531728	73/146 and 158/342	C -> T; G->S	PF3D7_1211900	G358S, with AF=0.5
HCR1, HCR2	chr5: 851823	103/103 and 246/246	T->C; M->V	PF3D7_0520800	
HCR1, HCR2	chr7: 605674	9/10 and 22/22	G->A; V->I	PF3D7_0713100	Depth is borderline. Pfmc-2TM: Maurer's
HCR1, HCR2	chr10: 1439013	41/41 and 88/88	G->A; V->I	PF3D7_1036400	liver: stage
HCR2	chr3: 136831	53/57	C -> G; Q->E	PF3D7_0302500	cytoadherence

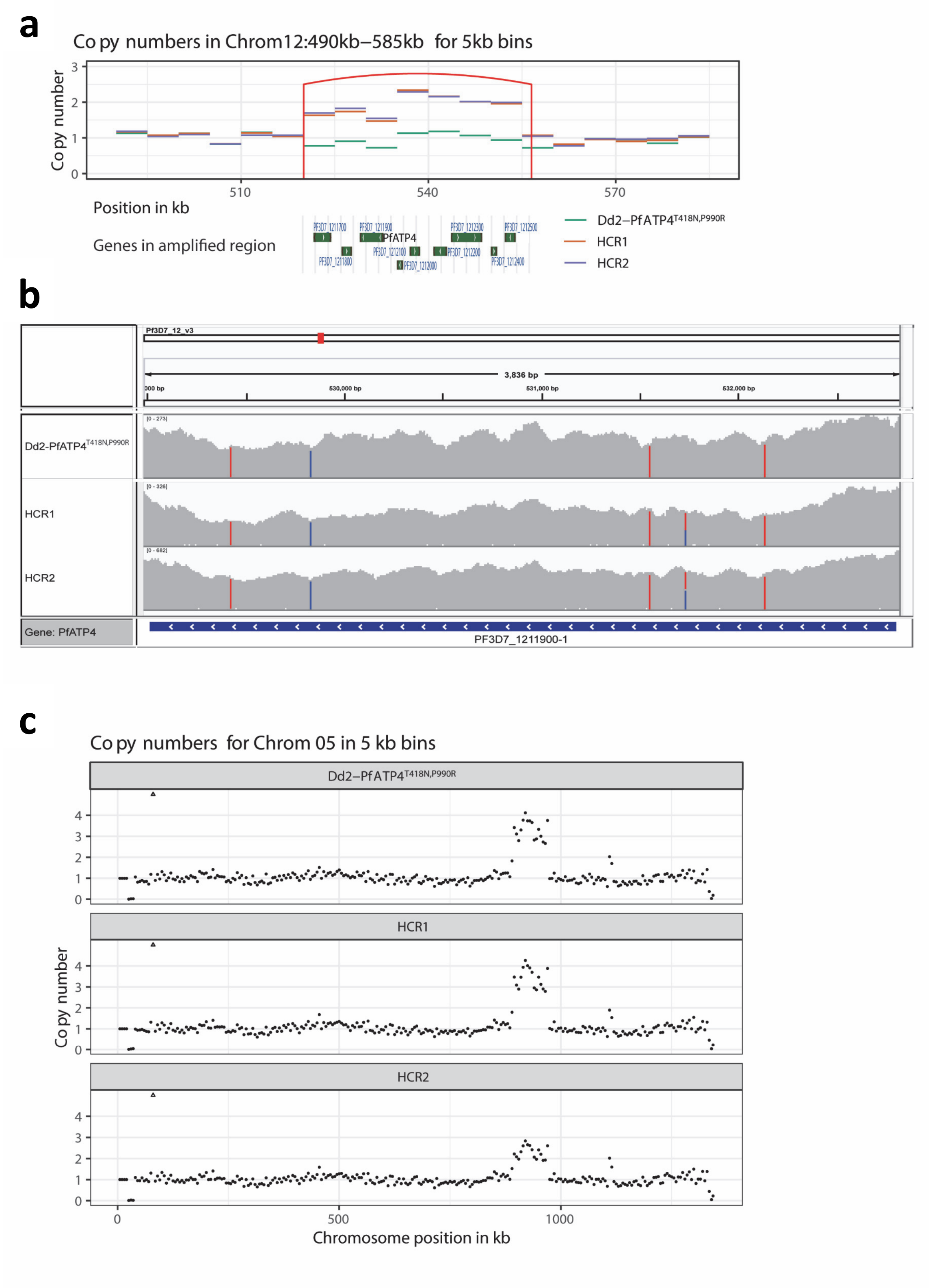

Supplementary Fig. 1. Whole-genome sequencing results for Dd2-PfATP4^{T418N,P990R}, HCR1 and HCR2. a. Duplicated region in chromosome 12 present in HCR1 (red) and HCR2 (blue) but not Dd2-PfATP4^{T418N,P990R} (green). Red vertical lines indicate breakpoint boundaries. **b**. Integrated Genome Viewer image of *pfatp4* region. The mutation giving rise to the G358S change in PfATP4 is in HCR1 and HCR2 with allele frequency of 0.5. The mutations giving rise to T418N and P990R are shown. Two mutations found in Dd2 parasites relative to 3D7 parasites (one synonymous (C669A in the *pfatp4* gene sequence; red bar closest to the right) and one giving rise to a G1128R change (red bar closest to the left)) are also shown. Bars are coloured by nucleotide if more than 25% of reads differ from reference. Red = T, blue = C. **c**. Differently-amplified region in chromosome 5, also detected by structural variant caller. The break ends are at 888 kb and 970 kb, including gene *pfmdr1*, location 955,955..963,095(+). Three or four copies are present in Dd2- PfATP4^{T418N,P990R} and HCR1, and one fewer in HCR2.

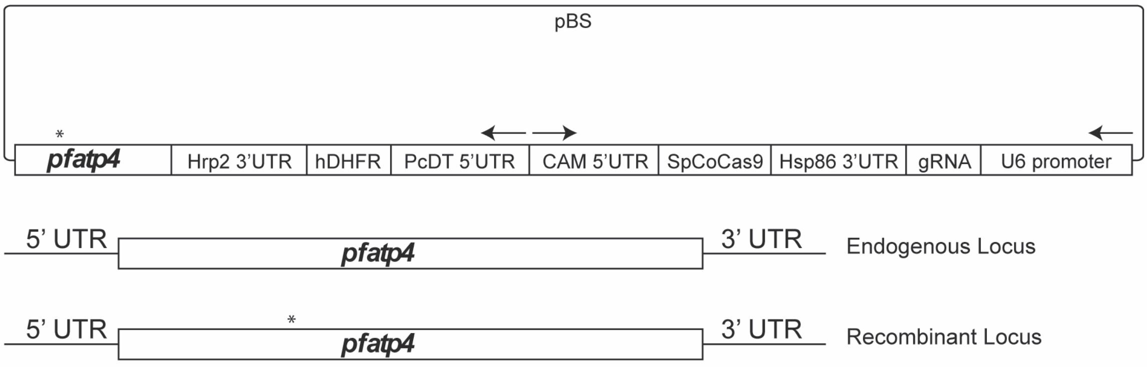

Supplementary Fig. 2. Strategy to introduce the PfATP4 G358S mutation into NF54 parasites. CRISPR-Cas9 was used to edit the endogenous *pfatp4* locus using an 'all-in-one' pDC2-based CRISPR-Cas9 plasmid (pDC2-cam-coSpCas9-U6-gRNA-hdhfr) described previously (3). Cas9 was derived from *Streptococcus pyogenes* (Sp), codon optimized (Co) for *P. falciparum*, and placed under the control of a *P. falciparum* calmodulin (CAM) promoter. The plasmid also contains a human dihydrofolate reductase (hDHFR) selectable marker driven by the *P. chabaudi dhfr-ts* (PcDT) promoter and a sequence encoding the guide RNA (gRNA) under a U6 promoter. The *pfatp4* donor has approximately 450 bp of homology flanking the G358S mutation.

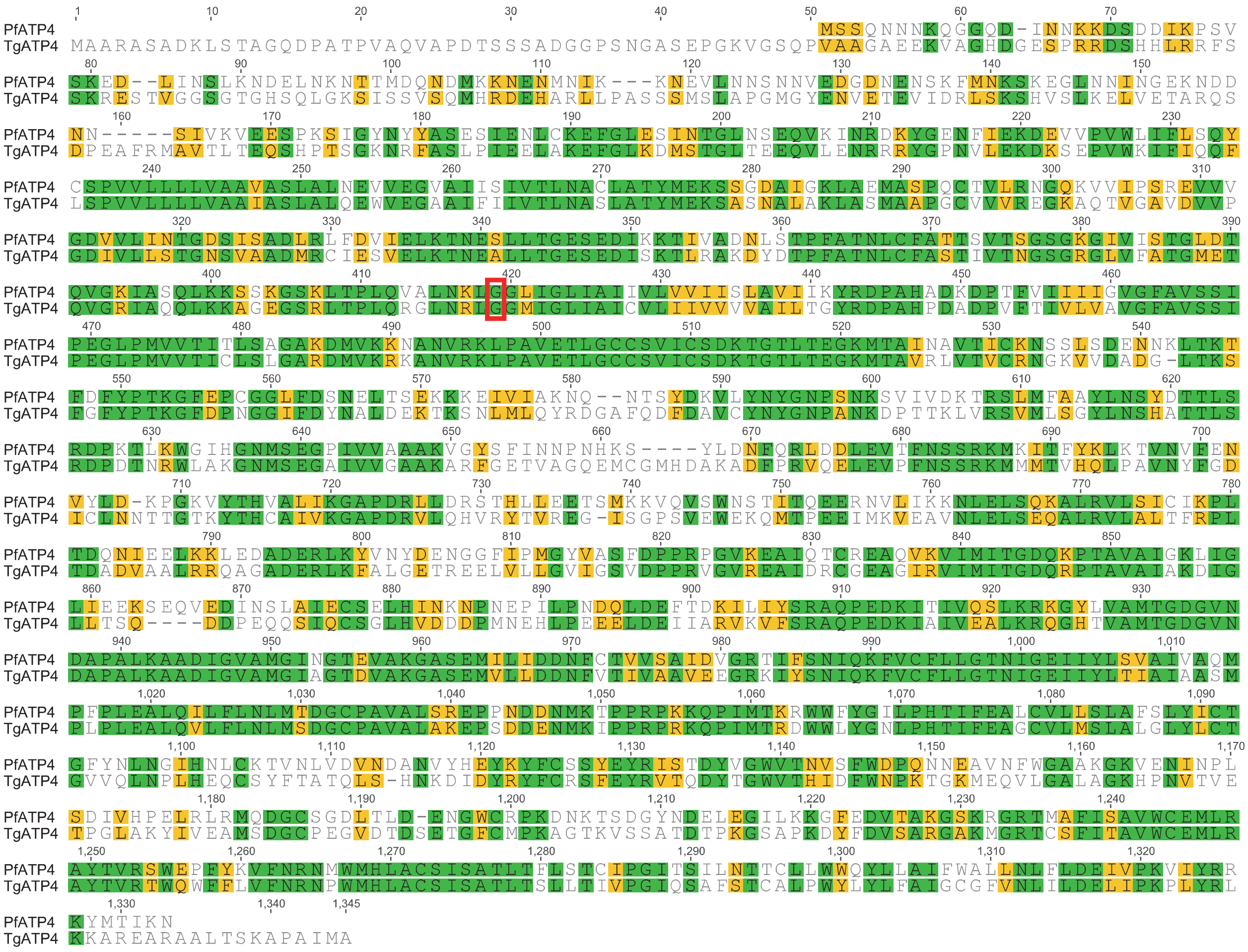

Supplementary Fig. 3. Sequence alignment of PfATP4 and TgATP4 and location of the G358 (PfATP4) and G419 (TgATP4) residues. The protein sequences for PfATP4 (PF3D7_1211900; https://plasmodb.org/plasmo/app/record/gene/PF3D7_1211900) and TgATP4 (TGGT1_278660; https://toxodb.org/toxo/app/record/gene/TGGT1_278660) were aligned in Geneious. Identical residues are shown in green and residues with similar chemical characteristics are shown in yellow. The G358 and G419 residues are shown in a red box.

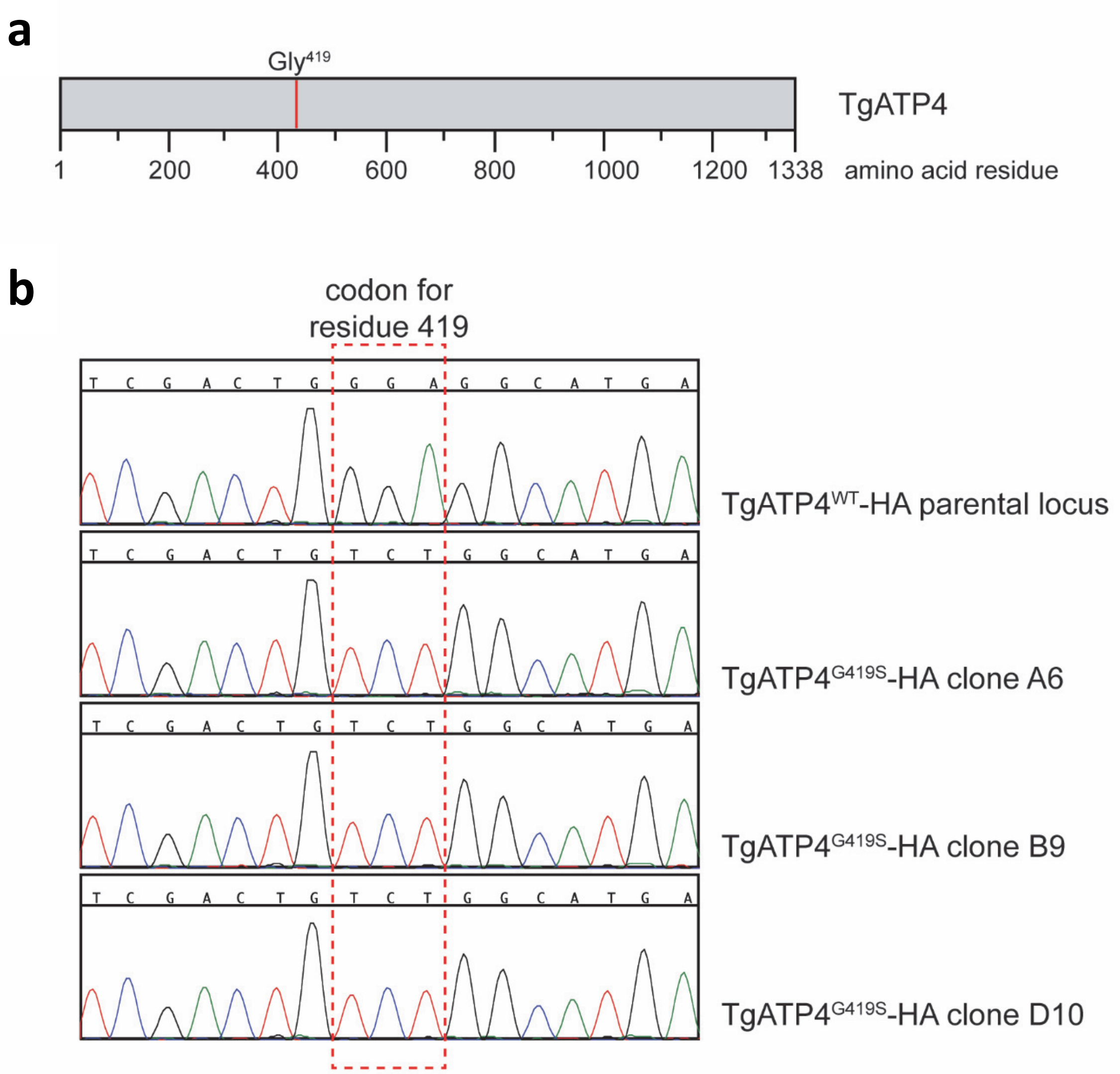

Supplementary Fig. 4. Generation of TgATP4^{G419S}-HA-expressing *T. gondii* parasite clones. a. Schematic of the amino acid sequence of TgATP4, drawn to scale and depicting the position of the glycine residue at amino acid position 419 (Gly^{419}). **b.** Sanger sequencing chromatograms of the region of the *tgatp4* genomic locus that encodes residue 419 in a parasite clone containing the 'wild type' parental locus (TgATP4^{WT}-HA; top) and in parasite clones containing the G419S-encoding mutations (TgATP4^{G419S}-HA, clones A6, B9 and D10). The red box depicts the codon that encodes residue 419, with a glycine (GGA) encoded in the parental strain, and a serine (TCT) encoded in the modified TgATP4^{G419S}-HA clones.

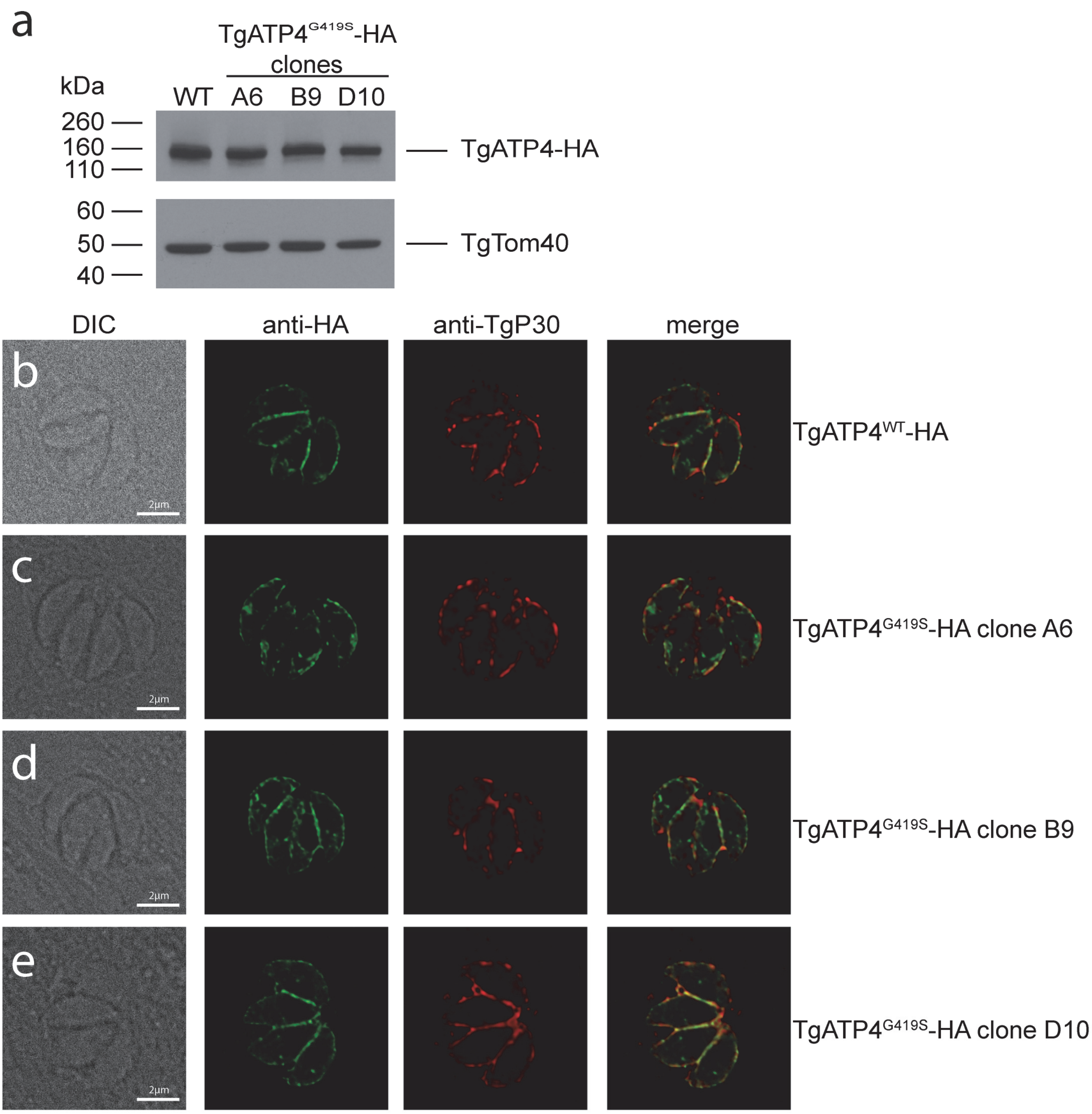

Supplementary Fig. 5. Expression and localisation of TgATP4^{G419S}-HA in *T. gondii* parasites. **a.** Western blot of TgATP4^{WT}-HA and TgATP4^{G419S}-HA expressing parasites, probed with anti-HA antibodies (top) and anti-TgTom40 antibodies as a loading control (bottom). **b-e.** Immunofluorescence assays of TgATP4^{WT}-HA (**b**) and TgATP4^{G419S}-HA expressing parasite clones (**c-e**) probed with anti-HA antibodies (green) and anti-TgP30 antibodies as a marker for the plasma membrane (red). Scale bars are 2 µm. DIC, differential interference contrast transmission images. The data are from a single experiment. Source data are provided as a Source Data file.

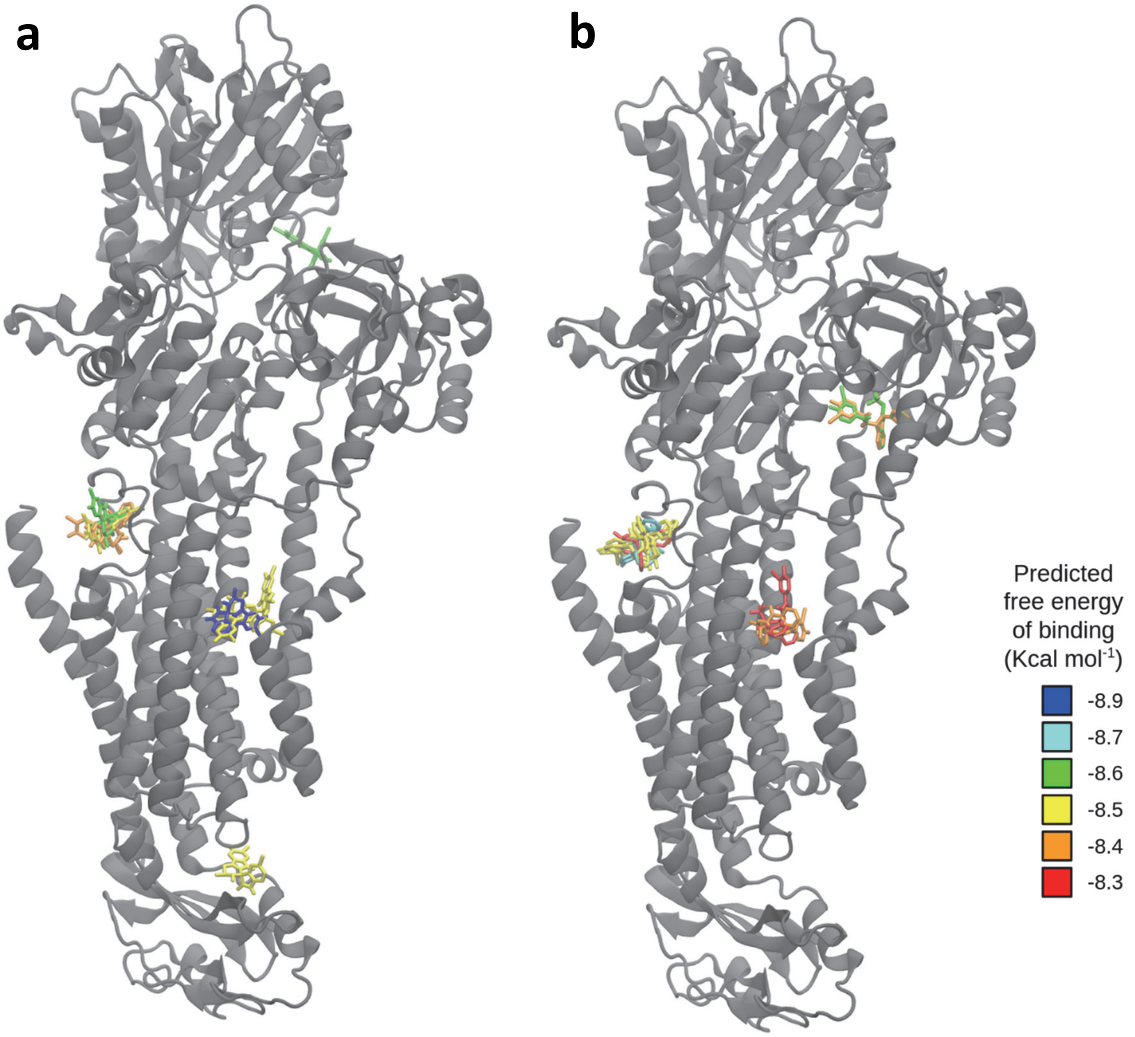

Supplementary Fig. 6. Binding sites for cipargamin and (+)-SJ733 found by AutoDock Vina when searching across the entire surface of the protein using the Open Source structure. The nine lowest predicted free energy poses of cipargamin (**a**) and (+)-SJ733 (**b**) are shown.

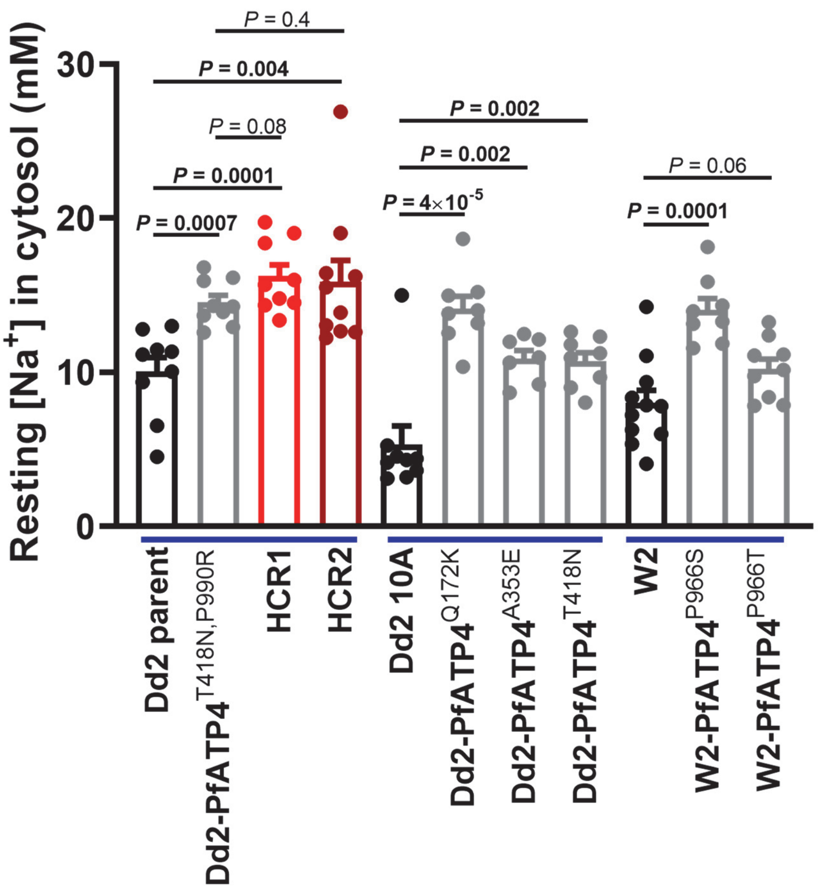

Supplementary Fig. 7. Resting cytosolic [Na^+] for parasite lines expressing wild-type or mutant variants of PfATP4. Data for three sets of lines are shown (see Methods for details of their origins), with the data for parental lines shown in black, those for parasites with low-level cipargamin resistance or for which the level of cipargamin resistance is not known shown in grey, and those for parasites with high-level cipargamin resistance shown in red. The measurements were performed with isolated trophozoite-stage parasites loaded with the Na^+-sensitive dye SBFI, and suspended in Physiological Saline Solution (pH 7.1) at 37˚C. The number of independent experiments performed was 7 for Dd2-PfATP4^{A353E}, 8 for Dd2-PfATP4^{Q172K}, Dd2-PfATP4^{T418N} and W2-PfATP4^{P966S}, 9 for Dd2 parent, Dd2-PfATP4^{T418N,P990R}, HCR1, Dd2 10A, W2-PfATP4^{P966T}, 10 for HCR2, and 11 for W2. The bars show the mean + SEM, and the symbols show the results obtained in individual experiments. The *P* values shown are from two-tailed unpaired t-tests. *P* values ≤ 0.05 indicate statistical significance and are shown in bold. Source data are provided as a Source Data file.

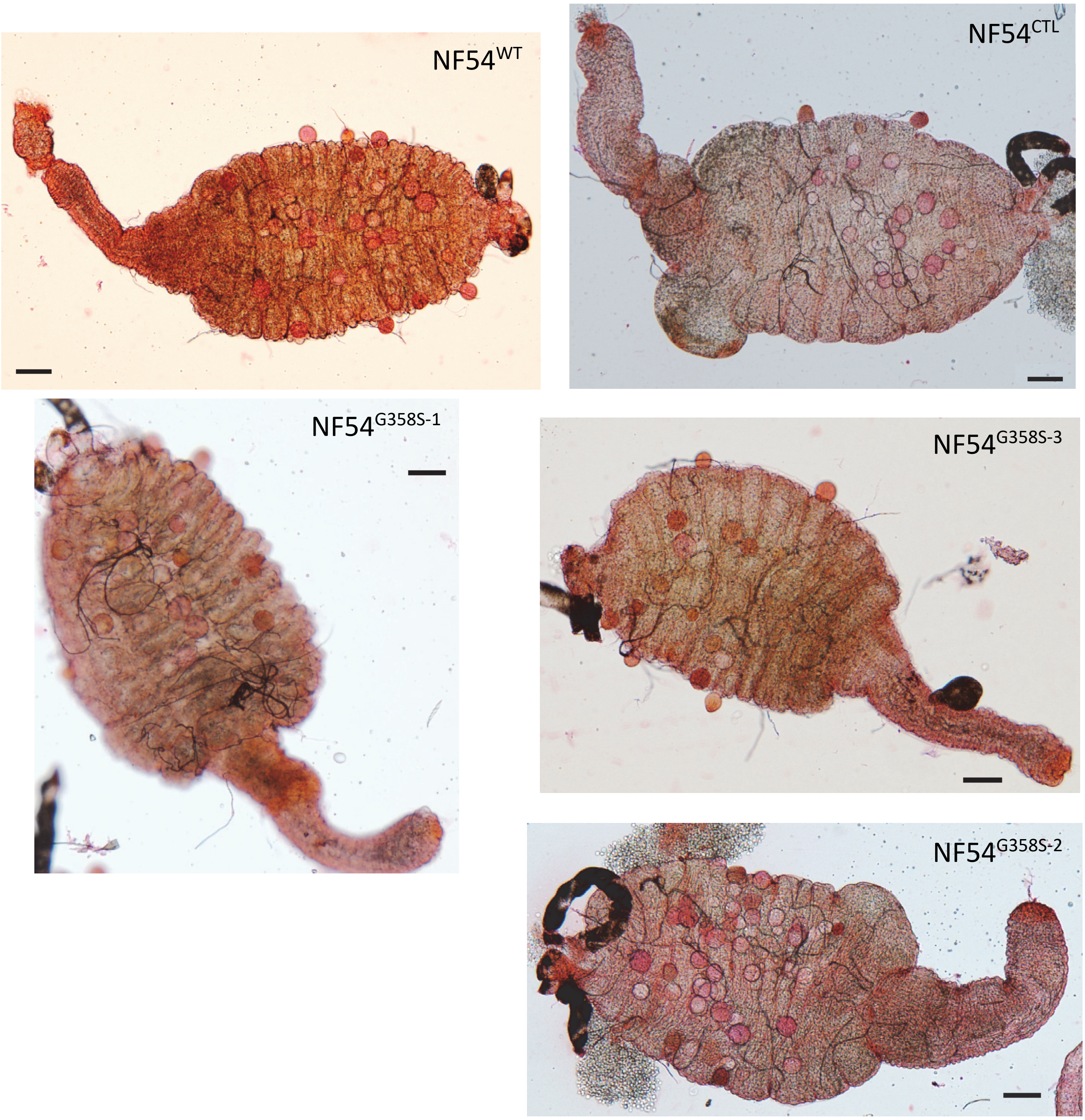

Supplementary Fig. 8. Representative images of *Anopheles stephensi* midguts infected with *P. falciparum* parasites. The midgut oocysts were stained with mercurochrome. The parasite line is shown in the top right-hand corner of each image. Scale bars = 100 μm.

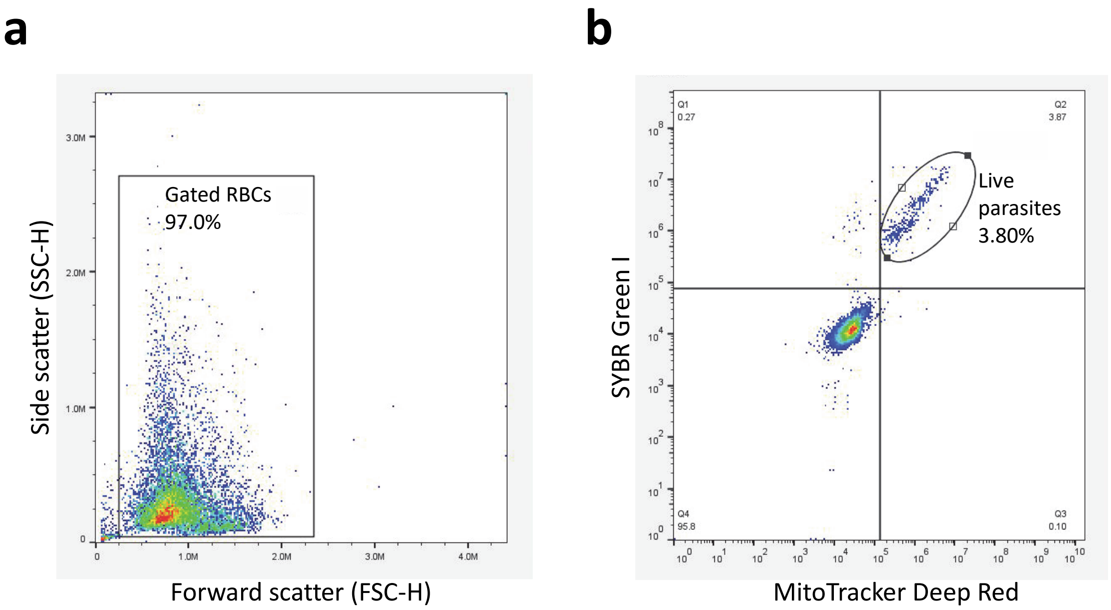

Supplementary Fig. 9. Gating strategy for flow cytometry-based quantification of *Plasmodium falciparum* NF54 asexual blood stage parasite proliferation in drug susceptibility assays. Intra-erythrocytic parasites were cultured at 0.3% parasitemia and 1% hematocrit for 72 hours in the presence of a range of drug concentrations that had been 2-fold serially diluted in duplicates along with drug-free controls. All assays were performed in culture media containing 10% O+ human serum. Cells were then labeled with 1X SYBR Green I (Invitrogen) and 200 nM MitoTracker Deep Red FM (Invitrogen) as nuclear stain and vital dyes, respectively. Parasite survival was assessed by flow cytometry on an Intellicyt iQue3 (Essen Bioscience). Between 9,000 and 15,000 events were counted per sample. Data show a control gating of untreated parasite cultures from a representative dose-response assay. **a.** Forward (FSC-H) and side scatter (SSC-H) gating of the total red blood cell (RBC) population, representing 97.0% of the total events counted. **b.** Gating strategy used to quantify viable intracellular parasites, which are positively stained by both MitoTracker Deep Red and SYBR Green I and appear as events in the upper right gate (live parasites, 3.80% parasitemia). Uninfected erythrocytes appear in the lower left gate as MitoTracker Deep Red and SYBR Green I negative populations. The gating strategy shown here was used to generate the data shown in Fig. 2c,f,i and Supplementary Table 5.

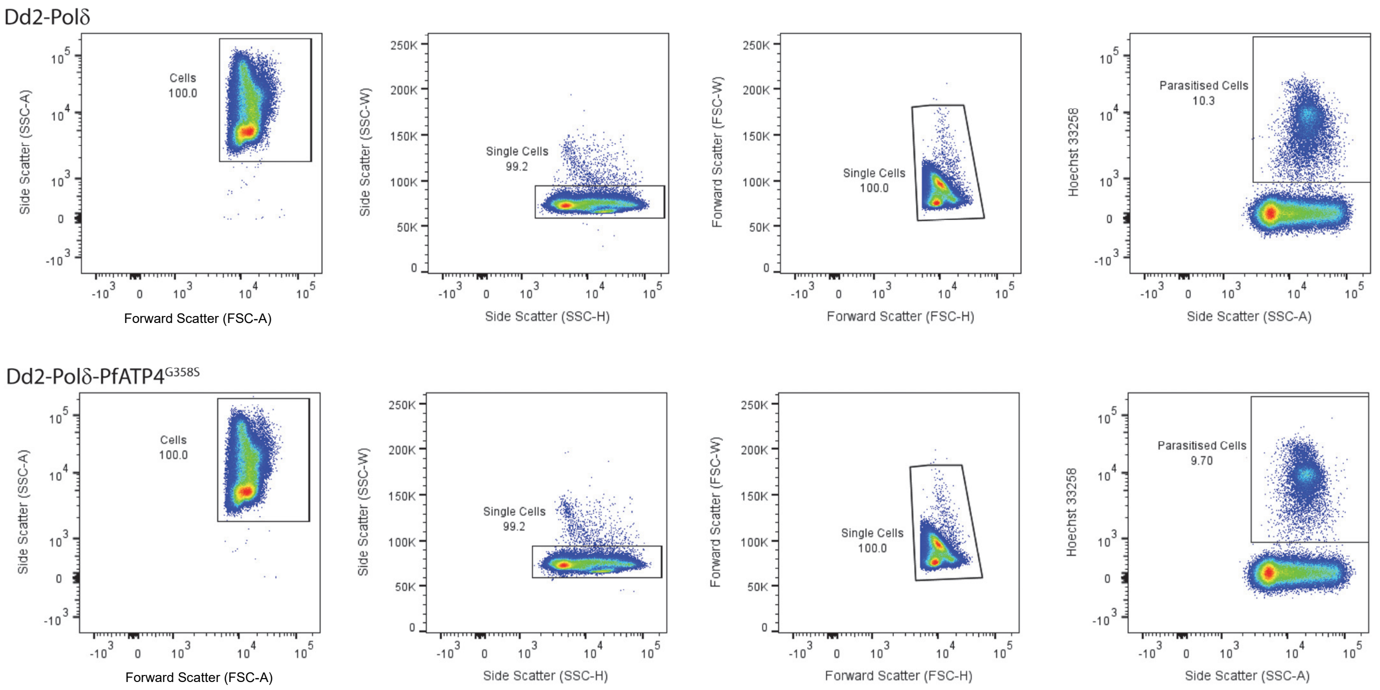

Supplementary Fig. 10. Gating strategy used to determine the parasitaemia in cultures of Dd2-Polδ and Dd2-Polδ-PfATP4^{G358S}. Erythrocytes (uninfected and infected with *P. falciparum*) were first gated in a plot of SSC-A versus FSC-A to exclude debris. Single cells were then gated in plots of SSC-W versus SSC-H and then FSC-W versus FSC-H, in both cases to exclude doublets and aggregates. Parasitised erythrocytes were then gated based on positivity for Hoechst 33258 staining (in a plot of Hoechst 33258 versus SSC-A). An example is shown for Dd2-Polδ (top) and Dd2-Polδ-PfATP4^{G358S}. The numbers inside the plots show the percentage of events that fall within the gate. The gating strategy illustrated here was used to generate the data shown in Fig. 7a.

Supplementary References

1. Rottmann, M., McNamara, C., Yeung, B. K., Lee, M. C., Zou, B., Russell, B., Seitz, P., Plouffe, D. M., Dharia, N. V., Tan, J., Cohen, S. B., Spencer, K. R., Gonzalez-Paez, G. E., Lakshminarayana, S. B., Goh, A., Suwanarusk, R., Jegla, T., Schmitt, E. K., Beck, H. P., Brun, R., Nosten, F., Renia, L., Dartois, V., Keller, T. H., Fidock, D. A., Winzeler, E. A. & Diagana, T. T. Spiroindolones, a potent compound class for the treatment of malaria. *Science* **329**, 1175-1180 (2010).
2. Lehane, A. M., Ridgway, M. C., Baker, E. & Kirk, K. Diverse chemotypes disrupt ion homeostasis in the malaria parasite. *Mol Microbiol* **94**, 327-339 (2014).
3. Murithi, J. M., Pascal, C., Bath, J., Boulenc, X., Gnadig, N. F., Pasaje, C. F. A., Rubiano, K., Yeo, T., Mok, S., Klieber, S., Desert, P., Jimenez-Diaz, M. B., Marfurt, J., Rouillier, M., Cherkaoui-Rbati, M. H., Gobeau, N., Wittlin, S., Uhlemann, A. C., Price, R. N., Wirjanata, G., Noviyanti, R., Tumwebaze, P., Cooper, R. A., Rosenthal, P. J., Sanz, L. M., Gamo, F. J., Joseph, J., Singh, S., Bashyam, S., Augereau, J. M., Giraud, E., Bozec, T., Vermat, T., Tuffal, G., Guillon, J. M., Menegotto, J., Salle, L., Louit, G., Cabanis, M. J., Nicolas, M. F., Doubovetzky, M., Merino, R., Bessila, N., Angulo-Barturen, I., Baud, D., Bebrevska, L., Escudie, F., Niles, J. C., Blasco, B., Campbell, S., Courtemanche, G., Fraisse, L., Pellet, A., Fidock, D. A. & Leroy, D. The antimalarial MMV688533 provides potential for single-dose cures with a high barrier to *Plasmodium falciparum* parasite resistance. *Sci Transl Med* **13**, eabg6013 (2021).
