# Supplementary material for: A G358S mutation in the Plasmodium falciparum Na+ pump PfATP4 confers clinically-relevant resistance to cipargamin
Source: Nat Commun. 2022 Sep 30;13:5746. doi: 10.1038/s41467-022-33403-9 (PMC9525273; doi:10.1038/s41467-022-33403-9)
Supplement: Supplementary file 3 — Reporting Summary [file 41467_2022_33403_MOESM3_ESM.pdf]

nature portfolio

Corresponding author(s): Adele Lehane
Last updated by author(s): Sep 15, 2022

Reporting Summary

Nature Portfolio wishes to improve the reproducibility of the work that we publish. This form provides structure for consistency and transparency in reporting. For further information on Nature Portfolio policies, see our Editorial Policies and the Editorial Policy Checklist.

Statistics

For all statistical analyses, confirm that the following items are present in the figure legend, table legend, main text, or Methods section.

n/a	Confirmed	
☐	☒	The exact sample size (n) for each experimental group/condition, given as a discrete number and unit of measurement
☐	☒	A statement on whether measurements were taken from distinct samples or whether the same sample was measured repeatedly
☐	☒	The statistical test(s) used AND whether they are one- or two-sided<br>*Only common tests should be described solely by name; describe more complex techniques in the Methods section.*
☒	☐	A description of all covariates tested
☒	☐	A description of any assumptions or corrections, such as tests of normality and adjustment for multiple comparisons
☐	☒	A full description of the statistical parameters including central tendency (e.g. means) or other basic estimates (e.g. regression coefficient) AND variation (e.g. standard deviation) or associated estimates of uncertainty (e.g. confidence intervals)
☐	☒	For null hypothesis testing, the test statistic (e.g. F, t, r) with confidence intervals, effect sizes, degrees of freedom and P value noted<br>*Give P values as exact values whenever suitable.*
☒	☐	For Bayesian analysis, information on the choice of priors and Markov chain Monte Carlo settings
☒	☐	For hierarchical and complex designs, identification of the appropriate level for tests and full reporting of outcomes
☒	☐	Estimates of effect sizes (e.g. Cohen's d, Pearson's r), indicating how they were calculated

Our web collection on statistics for biologists contains articles on many of the points above.

Software and code

Policy information about availability of computer code

Data collection	Flow cytometry: BD FACSDiva version 9.0<br>Generation of structural models: ColabFold version 1.3.0: Mirdita et al. 2022 Nat Methods<br>Docking: AutoDock Vina version 1.1.2: Trott & Olson 2010 J Comput Chem<br>Molecular dynamics: GROMACS 2021<br>SYBR Safe parasite proliferation assays: OPTIMA version 2.20R2 (BMG LABTECH)<br>Na(+) and ATPase assays: i-control version 1.12 (TECAN)<br>Cell volume measurements: Multisizer 4 software version 4.01 (Beckman)
Data analysis	Flow cytometry: FlowJo version 10 (FlowJo LLC)<br>Graphing and statistical analysis: SigmaPlot versions 11.0 and 14.0, GraphPad Prism versions 8 and 9<br>Microscope image deconvolution: SoftWoRx Suite version 2.0<br>Docking: VMD version 1.9.3: Humphrey et al. 1996 J Mol Graph<br>SYBR Safe parasite proliferation assays: MARS Data Analysis Software version 3.01 R2 (BMG LABTECH)<br><br>Analysis of whole genome sequencing data (HCR lines and parents):<br>Bowtie 2 version 2.2.5: Langmead & Salzberg 2012 Nat Methods<br>Picard tools MarkDuplicates version 2.2.2<br>SNVer version 0.5.3: Wei et al. 2011 Nucleic Acids Res<br>VarScan version 2.4: Koboldt et al. 2012 Genome Res<br>QDNaseq version 1.10.0: Scheinin et al. 2014 Genome Res<br>GRIDSS version 1.5: Cameron et al. 2017 Genome Res

Analysis of whole genome sequencing data (NF54-based lines):
Burrow-Wheeler Alignment (BWA) version 0.7.17
Samtools version 1.13
Picard MarkDuplicates (GATK version 4.2.2)
GATK BaseRecalibrator (GATK version 4.2.2)
GATK HaplotypeCaller (GATK version 4.2.2)
SnpEff version 4.3t: Cingolani et al. 2012 Fly (Austin)
BIC-Seq version 1.1.2: Xi et al. 2011 PNAS

For manuscripts utilizing custom algorithms or software that are central to the research but not yet described in published literature, software must be made available to editors and reviewers. We strongly encourage code deposition in a community repository (e.g. GitHub). See the Nature Portfolio guidelines for submitting code & software for further information.

Data

Policy information about availability of data

All manuscripts must include a data availability statement. This statement should provide the following information, where applicable:

- Accession codes, unique identifiers, or web links for publicly available datasets
- A description of any restrictions on data availability
- For clinical datasets or third party data, please ensure that the statement adheres to our policy

The whole genome sequencing data for this study have been deposited in the European Nucleotide Archive (ENA) at EMBL-EBI under accession numbers PRJEB53576 (HCR and parental lines; https://www.ebi.ac.uk/ena/browser/view/PRJEB53576) and PRJEB55457 (NF54-based lines; https://www.ebi.ac.uk/ena/browser/view/PRJEB55457). The structural models of PfATP4 generated in this study are available in GitHub (https://github.com/CorryLab/PfATP4_Colabfold-models). The 3D7 reference genomes used in the analyses are available in the PlasmoDB database. These were PlasmoDB-29_Pfalciparum3D7 for the HCR and parental lines (https://plasmodb.org/plasmo/app/downloads/release-29/Pfalciparum3D7/) and PlasmoDB-48_Pfalciparum3D7 for the NF54-based lines (https://plasmodb.org/plasmo/app/downloads/release-48/Pfalciparum3D7/). All other data supporting the findings of this study are available within the paper and Supplementary Information. Source data are provided with this paper.

Human research participants

Policy information about studies involving human research participants and Sex and Gender in Research.

Reporting on sex and gender	Plasmodium falciparum parasites were cultured in human red blood cells. Blood was provided by Australian Red Cross Lifeblood and the New York Blood Center and from healthy donors under an IRB-approved protocol at Johns Hopkins Hospital.
Population characteristics	No information available, as the donors of the human blood used in this study were anonymous.
Recruitment	Not applicable, as the donors of the human blood used in this study were anonymous.
Ethics oversight	The use of human blood in this study was approved by the Australian National University Human Research Ethics Committee (Protocol numbers 2011/266 and 2017/351) and the Johns Hopkins School of Medicine Institutional Review Board (Protocol number NA_00019050).

Note that full information on the approval of the study protocol must also be provided in the manuscript.

Field-specific reporting

Please select the one below that is the best fit for your research. If you are not sure, read the appropriate sections before making your selection.

☒ Life sciences ☐ Behavioural & social sciences ☐ Ecological, evolutionary & environmental sciences

For a reference copy of the document with all sections, see nature.com/documents/nr-reporting-summary-flat.pdf

Life sciences study design

All studies must disclose on these points even when the disclosure is negative.

Sample size	Sample size was not predetermined using statistical methods. At least three independent experiments were performed for all parasite proliferation assays and biochemical assays (e.g. ATPase assays and Na(+) assays). This is standard in the field, as it enables reproducibility to be checked and statistics to be performed. The exact number of biological replicates is stated in the relevant Figure legends. The western blot and immunofluorescence assay shown in Supplementary Fig. 5 were only performed once, however the expression and correct localisation of TgATP4 was also evidenced from biochemical assays that were performed at least three times.
Data exclusions	Data were only excluded in rare instances in which a technical error took place during the execution of an experiment.

Replication	All the experimental findings were found to be highly reproducible in biological replicates. To further check reproducibility, a number of the key experiments were performed by more than one researcher, with the results found to be highly reproducible in all cases. For example three different researchers performed Na(+) assays (Fig. 3) with P. falciparum parasites and all found that parasites with the G358S mutation in PfATP4 are highly resistant to cipargamin-mediated Na(+) dysregulation. Two different researchers performed ATPase assays (Fig. 4), with both researchers finding that the ATPase activity of G358S PfATP4 and G419S TgATP4 are resistant to inhibition by cipargamin and (+)-SJ733. Two different researchers showed that HCR parasites and Dd2-Pol(delta)-PfATP4(G358S) parasites display resistance to cipargamin, (+)-SJ733 and PA21A050 in parasite proliferation assays.
Randomization	In biochemical assays and parasite proliferation assays involving different compounds and/or parasite lines, there was randomization with respect to where these were positioned on the assay plates. Randomization was not relevant in other experiments, as separate cultures of different parasite lines were prepared and tested in the same manner.
Blinding	No blinding was performed in this study. The experiments were based on objective measurements that were not prone to investigator bias.

Reporting for specific materials, systems and methods

We require information from authors about some types of materials, experimental systems and methods used in many studies. Here, indicate whether each material, system or method listed is relevant to your study. If you are not sure if a list item applies to your research, read the appropriate section before selecting a response.

Materials & experimental systems

n/a	Involved in the study	
☐	☒	Antibodies
☐	☒	Eukaryotic cell lines
☒	☐	Palaeontology and archaeology
☐	☒	Animals and other organisms
☒	☐	Clinical data
☒	☐	Dual use research of concern

Methods

n/a	Involved in the study	
☒	☐	ChIP-seq
☐	☒	Flow cytometry
☒	☐	MRI-based neuroimaging

Antibodies

Antibodies used	Primary antibodies:<br>rat anti-HA, clone 3F10 (Sigma catalogue number 11867423001, diluted 1:500)<br>anti-TgTom40 (van Dooren et al. 2016 J Biol Chem, diluted 1:2000)<br>mouse anti-TgP30 clone TP3 (Abcam, ab8313, diluted 1:500)<br><br>Secondary antibodies:<br>horseradish peroxidase-conjugated goat anti-rat (Abcam, ab97057, diluted 1:5000)<br>horseradish peroxidase-conjugated goat anti-rabbit (Abcam, ab97051, diluted 1:10000)<br>donkey anti-rat AlexFluor 488 (Thermo Fisher Scientific, A-21208, diluted 1:500)<br>goat anti-rabbit AlexaFluor 546 (Thermo Fisher Scientific, A-11035, diluted 1:500)
Validation	The rat anti-HA antibody recognises a sequence of 9 amino acids (the HA tag, derived from the human influenza hemagglutinin protein and fused to other proteins of interest). The manufacturer states that its function was tested by western blot.<br>The anti-TgTom40 antibody was validated by an observed change in mass upon the introduction of an epitope tag into the TgTom40 locus (van Dooren et al. 2016 J Biol Chem, Figure 1C).<br>The anti-TgP30 antibody was used to mark the T. gondii plasma membrane in Supplementary Figure 5 and has been used for the same purpose in previous studies (e.g. Broncel et al. 2020 eLife, Zhou et al. 2021 iScience, Lehane et al. 2019 J Biol Chem). It has also been validated using immunofluorescence assays performed in the authors' laboratories through an inability to detect the protein upon knockout of the gene that encodes TgP30.

Eukaryotic cell lines

Policy information about cell lines and Sex and Gender in Research

Cell line source(s)	Plasmodium falciparum lines:<br>Transmission-competent NF54(WT) strain: BEI Resources, MRA-1000<br>Dd2-PfATP4(T418N,P990R) and its Dd2 parent: Rottmann et al. 2010 Science<br>Dd2-pol(delta): Kumpornsin et al. (bioRxiv preprint: https://doi.org/10.1101/2022.08.23.504974)<br>Dd2-B2-PfATP4(G358S), Dd2-B2 parent, W2-PfATP4(P966S), W2-PfATP4(P966T), W2 parent: Jimenez-Diaz et al. 2014 PNAS<br>Dd2 clone 10A, Dd2-PfATP4(T418N): Rosling et al. 2018 J Biol Chem<br><br>Toxoplasma gondii lines:<br>TgATP4-HA parasites: Lehane et al. 2019 J Biol Chem<br>TATi/(Delta)ku80 parasites: Sheiner et al. 2011 PLoS Pathog<br><br>Human foreskin fibroblasts were sourced from Holger Schlüter, Peter MacCallum Cancer Centre

Authentication	For genetically engineered lines, the presence of the desired genetic modification was confirmed in the studies listed above. Drug-selected lines were confirmed to have mutation(s) in PfATP4 in the relevant studies listed above.
Mycoplasma contamination	We tested the following Plasmodium falciparum lines for mycoplasma contamination during the course of the study by PCR using a mix of mycoplasma-specific primers (primer sequences are from Uphoff and Drexler 2002 Leukemia): HCR1, HCR2, Dd2-PfATP4(T418N,P990R) and its Dd2 parent. These cell lines all tested negative for mycoplasma contamination.<br><br>For the NF54-based lines, testing for mycoplasma was performed using a MycoAlert PLUS Mycoplasma Detection Kit (Lonza) prior to the start of drug assays. The lines tested negative for mycoplasma contamination.<br><br>For Dd2-pol(delta) and Dd2-pol(delta)-PfATP4(G358S), after the conclusion of the study parasites were cultured from freeze-downs made at various points during the study and tested for mycoplasma contamination by PCR. This revealed that the lines were initially negative for mycoplasma contamination, but that both became contaminated towards the end of the study. We therefore cured the lines of mycoplasma by culturing the parasites with Mycoplasma Removal Agent (MP Biomedicals) for 7 days. The MRA-treated parasites tested negative for mycoplasma contamination by PCR and we then performed further experiments with them (cytosolic Na(+), ATPase, and asexual growth assays), which in all cases gave rise to results entirely consistent with those obtained during the study.<br><br>Human foreskin fibroblasts used to culture Toxoplasma gondii were also tested for mycoplasma contamination by PCR and tested negative.
Commonly misidentified lines (See ICLAC register)	No commonly misidentified cell lines were used in this study.

Animals and other research organisms

Policy information about studies involving animals; ARRIVE guidelines recommended for reporting animal research, and Sex and Gender in Research

Laboratory animals	Anopheles stephensi mosquitoes Liston strain, 3 to 5 days post-emergence
Wild animals	The study did not involve wild animals.
Reporting on sex	Female Anopheles stephensi mosquitoes were used, as only females are involved in malaria parasite transmission.
Field-collected samples	The study did not involve samples collected from the field.
Ethics oversight	No ethical approval was required for the mosquito work and no other animals were used.

Note that full information on the approval of the study protocol must also be provided in the manuscript.

Flow Cytometry

Plots

Confirm that:

☒ The axis labels state the marker and fluorochrome used (e.g. CD4-FITC).

☒ The axis scales are clearly visible. Include numbers along axes only for bottom left plot of group (a 'group' is an analysis of identical markers).

☒ All plots are contour plots with outliers or pseudocolor plots.

☒ A numerical value for number of cells or percentage (with statistics) is provided.

Methodology

Sample preparation	For parasite proliferation assays with the NF54-based parasite lines and Dd2-B2-PfATP4(G358S) and its parent:<br>To detect surviving P. falciparum parasites, SYBR Green I and MitoTracker Deep Red FM (Thermo Fisher Scientific) were used as stains for DNA and cell viability, respectively.<br><br>For asexual growth comparisons between Dd2-Pol(delta) and Dd2-Pol(delta)-PfATP4(G358S):<br>P. falciparum infected erythrocytes suspended in pH 7.4 Physiological Saline Solution were stained with Hoechst 33258.
Instrument	For parasite proliferation assays with the NF54-based parasite lines and Dd2-B2-PfATP4(G358S) and its parent:<br>Accuri C6 (BD Biosciences) and HyperCyt autosampler (Intellicyt)<br><br>For asexual growth comparisons between Dd2-Pol(delta) and Dd2-Pol(delta)-PfATP4(G358S):<br>BD LSR II
Software	For parasite proliferation assays with the NF54-based parasite lines and Dd2-B2-PfATP4(G358S) and its parent:<br>FlowJo version 10 (FlowJo LLC.)

For asexual growth comparisons between Dd2-Pol(delta) and Dd2-Pol(delta)-PfATP4(G358S):
BD FACSDiva version 9.0 and FlowJo version 10 (FlowJo LLC.)

Cell population abundance

For parasite proliferation assays with the NF54-based parasite lines and Dd2-B2-PfATP4(G358S) and its parent:
Flow cytometry was used to quantify parasite survival in the presence of various drugs at a range of different concentrations. The parasitaemia (% of red blood cells containing live parasites) ranged from 0% to 6% in different samples. Cells were not sorted for these assays.

For asexual growth comparisons between Dd2-Pol(delta) and Dd2-Pol(delta)-PfATP4(G358S):
Flow cytometry was used to quantify the percentage of red blood cells that were parasitised, which ranged from 6 to 19% in the different samples. Cells were not sorted for these assays.

Gating strategy

For parasite proliferation assays with the NF54-based parasite lines and Dd2-B2-PfATP4(G358S) and its parent:
The gating strategy has been described previously (Straimer et al. 2015 Science). Red blood cells (uninfected and infected with P. falciparum) were first gated using forward scatter (FSC) and side scatter (SSC) channels. Red blood cells infected with live parasites were observed as positive events in the FL1 (SYBR Green I) and FL4 (MitoTracker Deep Red) channels (upper right quadrant gate of plot).

For asexual growth comparisons between Dd2-Pol(delta) and Dd2-Pol(delta)-PfATP4(G358S):
Red blood cells (uninfected and infected with P. falciparum) were first gated in a plot of SSC-A versus FSC-A (to exclude debris). Single cells were then gated in plots of SSC-W versus SSC-H and then FSC-W versus FSC-H (to exclude doublets and aggregates). Parasitised erythrocytes were then gated based on positivity for Hoechst 33258 staining (in a plot of Hoechst 33258 versus SSC-A).

☒ Tick this box to confirm that a figure exemplifying the gating strategy is provided in the Supplementary Information.
